# Coconut (*Cocos nucifera* (L.)) Water Improves Glucose Uptake with Concomitant Modulation of Antioxidant and Purinergic Activities in Isolated Rat Psoas Muscles

**DOI:** 10.3390/plants13050665

**Published:** 2024-02-28

**Authors:** Ochuko L. Erukainure, Chika I. Chukwuma

**Affiliations:** 1 Laser Research Centre, Faculty of Health Sciences, University of Johannesburg, Doornfontein 2028, South Africa; 2 Centre for Quality of Health and Living, Faculty of Health and Environmental Sciences, Central University of Technology, Bloemfontein 9301, South Africa; chykochi@yahoo.com

**Keywords:** coconut water, glucose uptake, oxidative stress, type 2 diabetes

## Abstract

The present study investigated the effect of coconut water on glucose uptake and utilization, and metabolic activities linked to hyperglycemia in isolated rat psoas muscles. Coconut water was subjected to in vitro antioxidant and antidiabetic assays, which cover 2,2′-diphenyl-1-picrylhydrazyl (DPPH) scavenging activity, ferric reducing antioxidant power (FRAP), and inhibition of α-glucosidase and α-amylase activities. Psoas muscles were isolated from male Sprague Dawley rats and incubated with coconut water in the presence of glucose. Control consisted of muscles incubated with glucose only, while normal control consisted of muscles not incubated in coconut water and/or glucose. The standard antidiabetic drug was metformin. Incubation with coconut water led to a significant increase in muscle glucose uptake, with concomitant exacerbation of glutathione level, and SOD and catalase activities, while suppressing malondialdehyde level, and ATPase and E-NTDase activities. Coconut water showed significant scavenging activity against DPPH, and significantly inhibited α-glucosidase and α-amylase activities. LC-MS analysis of coconut water revealed the presence of ellagic acid, butin, quercetin, protocatechuic acid, baicalin, and silibinin. Molecular docking analysis revealed potent molecular interactions between the LC-MS-identified compounds, and AKT-2 serine and PI-3 kinase. These results indicate the potential of coconut water to enhance glucose uptake, while concomitantly improving antioxidative and purinergic activities. They also indicate the potential of coconut water to suppress postprandial hyperglycemia. These activities may be attributed to the synergistic effects of the LC-MS-identified compounds.

## 1. Introduction

Type 2 diabetes (T2D) ranks among the most prevalent type of diabetes mellitus (DM) as it accounts for over 90% of all diabetes types, and is thus a major contributor to DM-related morbidities and mortalities [1]. It is characterized by hyperglycemia arising from insulin resistance and pancreatic β-cell dysfunction. Persistent hyperglycemia has been linked to increased production of free radicals and reactive oxygen species (ROS), which surpasses the body’s antioxidant defense system, leading to oxidative stress [2,3].

Skeletal muscles play a major role in glucose metabolism and insulin function, as over 80% of the postprandial circulatory glucose uptake is attributed to them [4,5]. Desensitization of skeletal muscles to insulin secreted by the pancreas to stimulate glucose uptake results in insulin resistance and hyperglycemia [4]. Impaired muscle glucose uptake has been associated with a metabolic switch to free fatty acid (FFA) oxidation for energy generation, with concomitant generation of oxidative stress, thus further contributing to suppressed insulin sensitivity [6,7]. 

Impaired muscle glucose uptake has been implicated in the pathophysiology of T2D, and is a major therapeutic target in the treatment of T2D. Some antidiabetic drugs such as the biguanides (e.g., metformin) improve glucose homeostasis by stimulating muscle glucose uptake, while concomitantly suppressing FFA oxidation and oxidative stress [8,9]. However, the cost and side effects associated with synthesized drugs have led to the continuous search for affordable alternatives with fewer side effects. These searches have led to a paradigm shift to natural products, which are readily affordable with fewer side effects.

Coconut water is a natural beverage found in the endosperm of coconut palm, *Cocos nucifera*, from the family of Arecaceae (Palmae). Coconut water is regarded as a functional food and/or nutraceutical with reported nutritional and medicinal properties. Its reported medicinal properties include antilipemic, hepatoprotective cardioprotective, and antihypertensive activities [10]. Its antidiabetic properties have been well reported and have been attributed to its ability to suppress blood glucose level, improve glucose tolerance, and restore pancreatic morphology [11,12]. Its ability to protect against diabetic retinopathy has been attributed to its modulation of antioxidant and anti-inflammatory activities and improvement in total retina thickness and thickness of the retinal nuclear layer, while increasing the number of neurons in the ganglion cell layer [13]. Coconut water has also been reported for its ability to improve insulin synthesis, decrease glycosylated hemoglobin levels, improve weight gain, and modulate the L-arginine-nitric oxide pathway in diabetic rats [14,15]. These activities have been attributed to the phytochemical properties of coconut water, which include flavonoids, phytates, oxalates, and alkaloids [16].

Although the hypoglycemic activities of coconut water have been reported, there is still a dearth of scientific reports on the ability of coconut water to stimulate skeletal muscle glucose uptake and utilization, and modulate activities linked to hyperglycemia. Thus, the present study was aimed at investigating the effect of coconut water on glucose uptake and utilization, and metabolic activities linked to hyperglycemia in isolated rat psoas muscles.

## 2. Results

**Figure 1:**
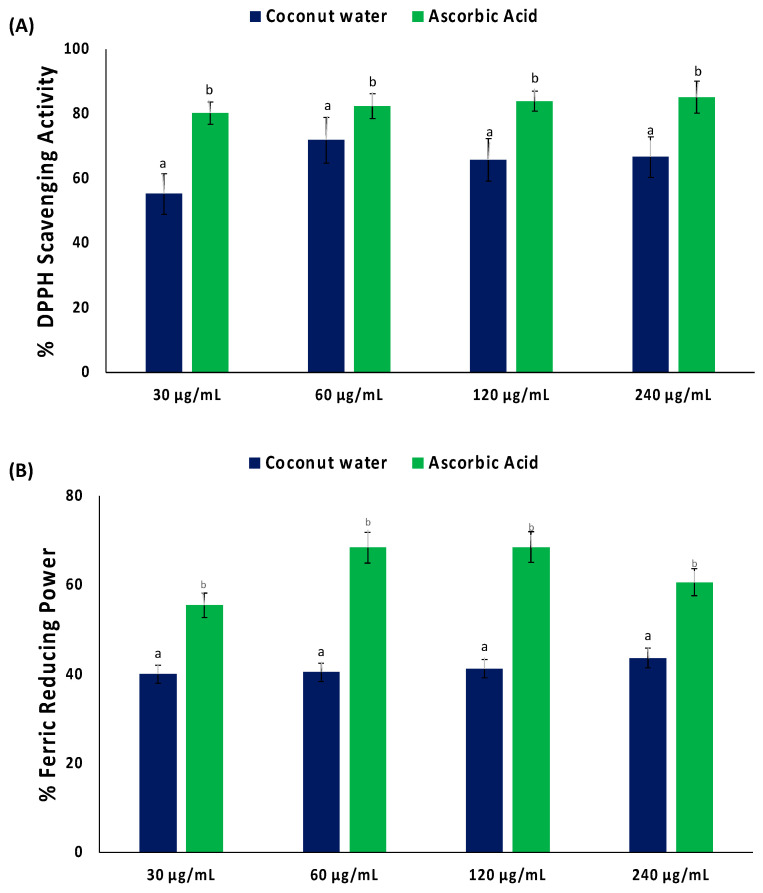
(**A**) DPPH scavenging activity and (**B**) ferric reducing power of coconut water. Values = mean ± SD; n = 3. ^a,b^ Values with different letter above the bars for a given extract are significantly different from each other (*p* < 0.05, Tukey’s HSD multiple range post hoc test, IBM SPSS for Windows).

As shown in Figure 1A, coconut water significantly (*p* < 0.05) scavenged DPPH radical, with an IC_50_ value of 34.09 μg/mL (Table 1). Coconut water also displayed a reducing power activity, as depicted by its ability to reduce Fe^3+^ to Fe^2+^ (Figure 1B). However, the activity was not potent, as depicted by its IC_50_ value of >1000 μg/mL (Table 1).

**Figure 2:**
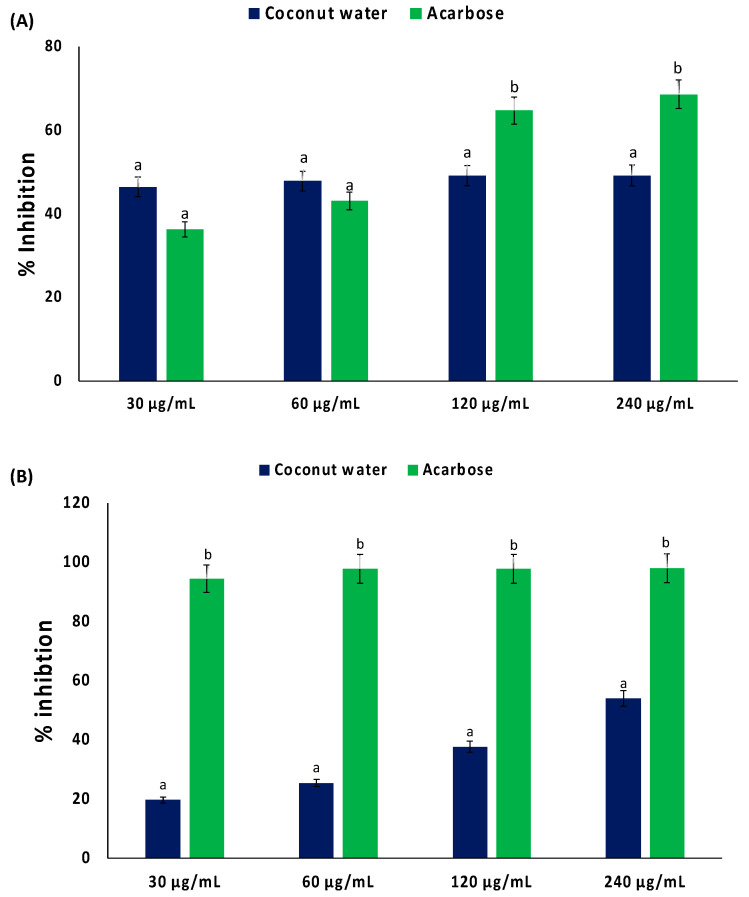
Inhibitory effect of coconut water on (**A**) α-glucosidase and (**B**) α-amylase activities. Values = mean ± SD; n = 3. ^a,b^ Values with different letter above the bars for a given extract are significantly different from each other (*p* < 0.05, Tukey’s HSD multiple range post hoc test, IBM SPSS for Windows).

As shown in Figure 2A,B, coconut water significantly (*p* < 0.05) inhibited the activities of α-glucosidase and α-amylase, with IC_50_ values of 338.27 and 219.73 μg/mL, respectively (Table 1). These activities were not dose dependent.

**Figure 3:**
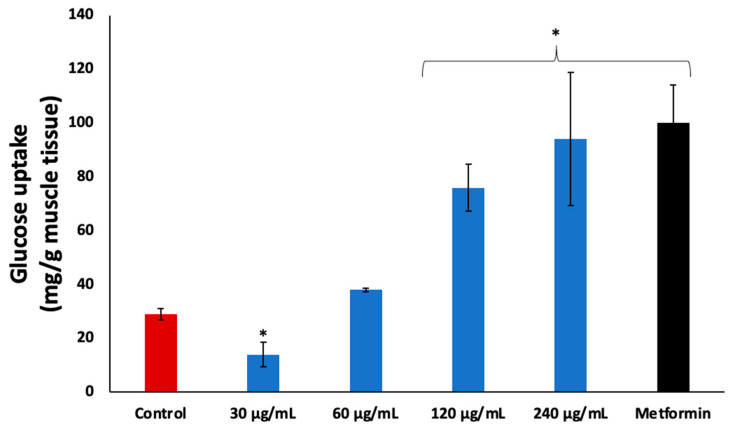
Effect of coconut water on glucose uptake in isolated psoas muscle. Values = mean ± SD; n = 3. * Statistically significant to normal and control, respectively (*p* < 0.05, Tukey’s HSD multiple range post hoc test, IBM SPSS for Windows). **Control**: muscles incubated with glucose only.

Incubation of psoas muscle with coconut water in the presence of glucose led to significant (*p* < 0.05) glucose uptake, as shown in Figure 3. The activity was dose dependent with increasing concentrations, and compared favorably to the standard drug, metformin. 

**Figure 4:**
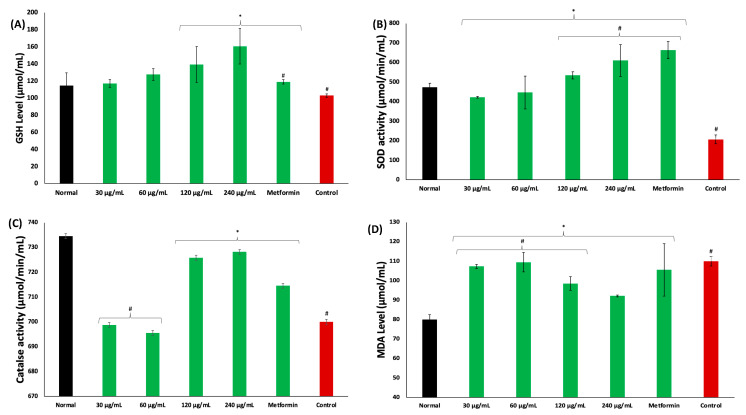
Effect of coconut water on (**A**) GSH level; (**B**) SOD activity; (**C**) catalase activity; and (**D**) MDA level. Values = mean ± SD; n = 3. *, # Statistically significant to normal and control, respectively (*p* < 0.05, Tukey’s HSD multiple range post hoc test, IBM SPSS for Windows). **Normal**: muscles not subjected to incubation; **Control**: muscles incubated with glucose only.

Incubation of psoas muscles in glucose led to significant (*p* < 0.05) depletion in GSH level, and SOD and catalase activities, while concomitantly elevating MDA level, as shown in Figure 4A–D, respectively. These levels and activities were significantly reversed in muscles incubated with coconut water, as depicted by elevated GSH level and SOD and catalase activities, and decreased MDA level. These activities were dose dependent and compared favorably with metformin.

**Figure 5:**
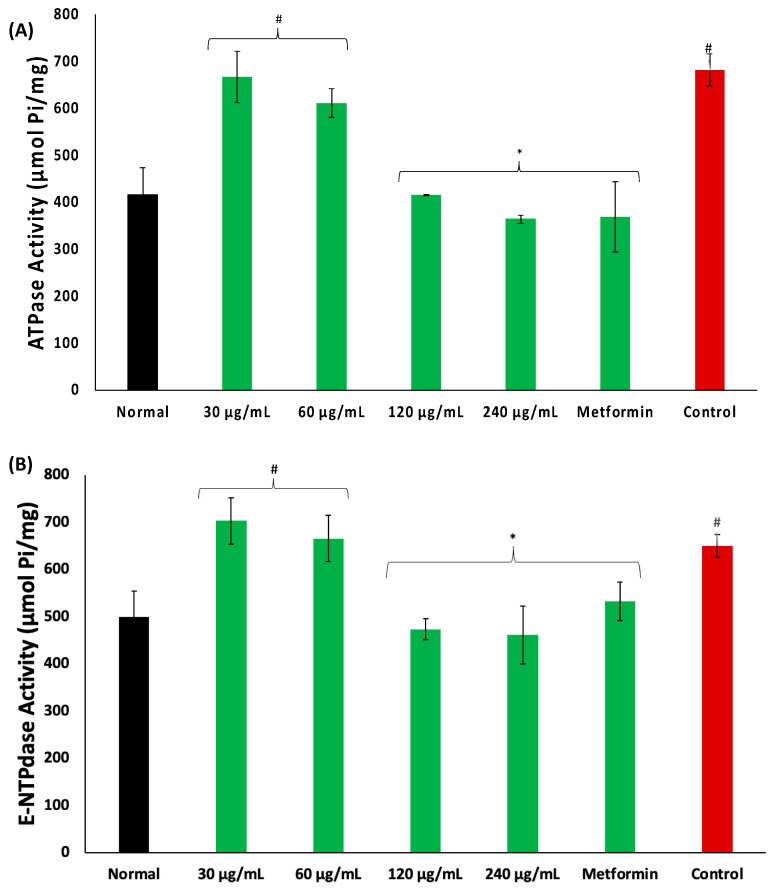
Effect of coconut water on (**A**) ATPase and (**B**) E-NTPDase activities. Values = mean ± SD; n = 3. *, # Statistically significant to normal and control, respectively (*p* < 0.05, Tukey’s HSD multiple range post hoc test, IBM SPSS for Windows). **Normal**: muscles not subjected to incubation; **Control**: muscles incubated with glucose only.

There was an increase in the activities of ATPase and E-NTPDase in psoas muscles incubated in glucose, as depicted in Figure 5A,B, respectively. These activities were significantly (*p* < 0.05) and dose-dependently reversed in muscles incubated with coconut water.

**Figure 6:**
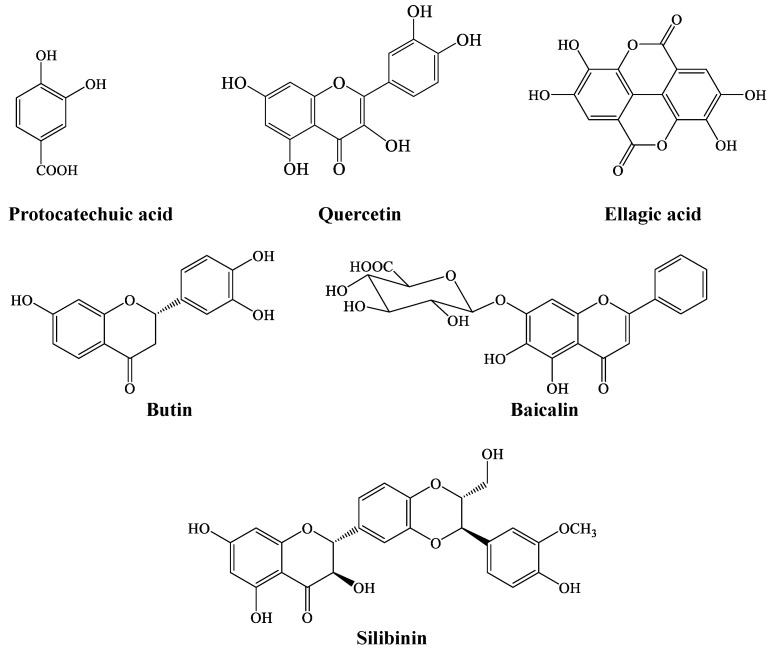
Chemical structure of LC-MS-identified compounds in coconut water.

LC-MS analysis of coconut water revealed the presence of phenolic compounds, which are ellagic acid, butin, quercetin, protocatechuic acid, baicalin, and silibinin, as shown in Figure 6.

**Figure 7:**
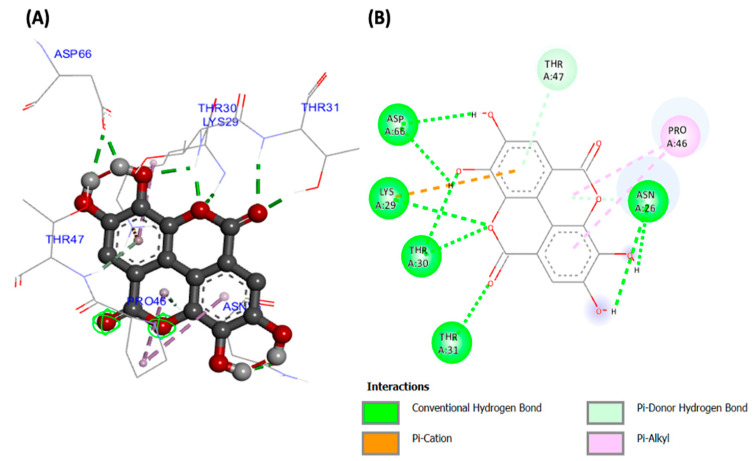
(**A**) Three- and (**B**) two-dimensional presentations of molecular interaction of ellagic acid with AKT-2 serine (PDB ID: 3DOE).

**Figure 8:**
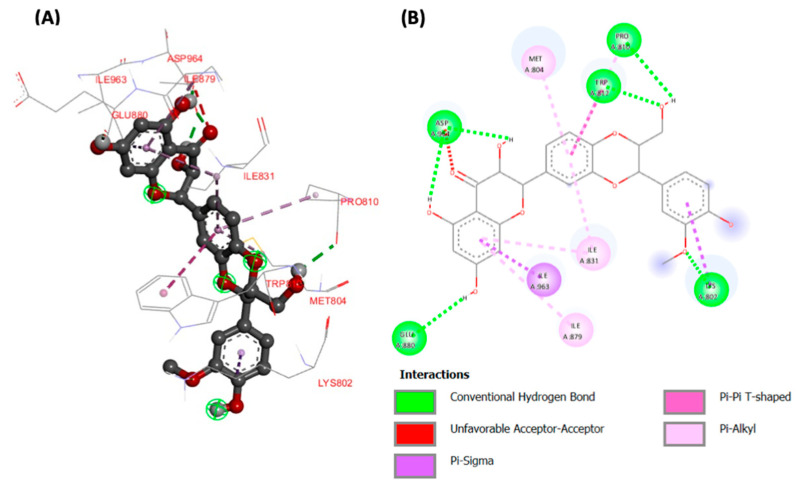
(**A**) Three- and (**B**) two-dimensional presentations of molecular interaction of silibinin with and PI-3 kinase (PDB ID: 7JWZ).

Molecular docking analyses revealed potent molecular interactions of the identified compounds with AKT-2 serine (3DOE) and PI-3 kinase (7JWZ), as depicted by their free energies (Table 2). The highest binding affinities were observed for ellagic acid with AKT-2 serine (Figure 7), and silibinin with PI-3 kinase (Figure 8), with free energies of −8.99 and −9.03 kcal/mol, respectively.

## 3. Discussion

The continuous search for affordable treatments for T2D has led to an increasing interest in natural products from medicinal plants. Medicinal plants have been used from time immemorial to treat several diseases including T2D and form major part of the traditional medicine system [17,18]. Although the hypoglycemic properties of these plants have been demonstrated, there still remains a dearth of data on the mechanism by which they bring about this effect. In the present study, we investigated the antidiabetic properties of coconut water by investigating its potential to stimulate glucose uptake and modulate activities linked to glucose homeostasis in isolated psoas muscles, as well as inhibit key enzymes linked to glucose digestion.

The inhibition of glucose-metabolizing enzymes has been reported as one of the major antidiabetic mechanisms [19]. These enzymes catalyze the hydrolysis of dietary carbohydrate to glucose, leading to a postprandial rise in blood glucose level. Thus, the inhibition of α-glucosidase and α-amylase activities by coconut water (Figure 2A,B, and Table 1) indicates a suppressive potential against postprandial hyperglycemia. Previous reports on the inhibitory effect of coconut water on these enzymes related to its extracted polysaccharides [20]. Results from the present study corroborate several reports about the inhibitory effect of plants and their phytochemicals on glucose-metabolizing enzymes [21,22,23].

Muscle glucose uptake has also been reported as a major mechanism of maintaining postprandial glucose homeostasis. In the fed state, the pancreas secretes insulin which stimulates muscle glucose uptake, which the muscles utilize as fuel substrate for energy generation [24,25]. Impairment of muscle glucose uptake arising from insulin resistance has been reported as a part of the pathophysiology of T2D, as it contributes to hyperglycemia [26]. The increased glucose uptake in psoas muscles incubated with coconut water (Figure 3) therefore indicates a glucose uptake stimulatory effect. It further suggests the ability of coconut water to suppress postprandial hyperglycemia, and thus maintain glucose homeostasis. This activity may contribute to the reported hypoglycemic properties of coconut water in diabetic rats [14,15,27].

Exacerbated muscle oxidative stress has been linked to impaired muscle glucose uptake and has been attributed to increased generation of free radicals and ROS, which suppresses the endogenous antioxidant defense system [28,29]. This corroborates with the suppressed GSH level and SOD and catalase activities, and elevated MDA level in psoas muscle incubated in only glucose (control) (Figure 4A–D). Similar activities and levels have been reported in impaired glucose uptake [25,29]. The exacerbated GSH level and SOD and catalase activities, and suppressed MDA level, in psoas muscles incubated in coconut water indicates an antioxidative effect. This effect also corroborates the scavenging and reducing power activities of coconut water (Figure 1A,B, and Table 1), thus indicating the ameliorative effect of coconut water against oxidative stress, which is among the major pathogenesis and pathophysiology of T2D [30,31]. This corroborates previous reports on the antioxidant properties of coconut water [14,32,33].

Alterations in the purinergic enzyme activities of muscles have been linked to impaired muscle dysfunctions including glucose uptake, as these enzymes are involved in the hydrolysis of adenosine triphosphate (ATP) and adenosine monophosphate (AMP) to generate adenine [34,35]. Adenine is an endogenous signaling nucleotide with reported physiological roles in glucose and bioenergetic homeostasis [36]. The exacerbated ATPase and E-NTPadase activities in psoas muscles incubated with only glucose (Figure 5A,B) indicate a suppressed level of ATP, thus suggesting limited availability of the energy substrates. This corroborates previous reports on increased muscle purinergic enzyme activities in impaired glucose uptake [24,29,35]. The suppressed activities of these enzymes in psoas muscles incubated with coconut water, therefore, indicate an availability of ATP for the generation of adenine and muscle biofunctions including glucose uptake. 

These antioxidative, antidiabetic, and purinergic activities can be attributed to the synergistic effect of the LC-MS-identified compounds (Figure 6). These compounds have been well reported for their potent antioxidant and antidiabetic activities [37,38,39,40,41,42]. These activities can be can be attributed to the phenolic structure, which consists of hydroxyl groups and benzene rings. The hydroxyl groups interact with the Π-electrons of the benzene ring, causing the radical to delocalize, and they also donate one electron to the free radical to produce a stable product [43]. 

The potent molecular interactions of the identified compounds with AKT-2 serine and PI-3 kinase (Figure 7 and Figure 8, and Table 2) portray molecular mechanisms by which the compounds may synergistically improve insulin sensitivity and stimulate muscle glucose uptake. AKT-2 serine plays a key role in insulin-stimulated glucose uptake in muscles by upregulating GLUT4 translocation [44,45]. PI-3 kinase has been reported for its role in insulin signaling and insulin-mediated translocation of GLUT4, thereby driving muscle glucose uptake [46,47].

## 4. Materials and Methods

### 4.1. Coconut Water

Mature coconuts were bought from a local fruit market in Lagos, Nigeria. The coconuts were dehusked and cracked, and the water collected into beakers. The water was frozen overnight at −80 °C, before freeze drying. The freeze-dried samples were collected into vials and stored at −4 °C.

Using a stock solution of 1 mg/mL of the freeze-dried sample, various concentrations of 15, 30, 60, 120, and 240 µg/mL were prepared for in vitro and ex vivo investigations.

### 4.2. In Vitro Antioxidant Activity

The sample was subjected to in vitro antioxidant analyses which cover 2,2′-diphenyl-1-picrylhydrazyl (DPPH) scavenging activity and ferric reducing antioxidant power (FRAP) [48,49]. 

Briefly, 100 μL of the sample’s concentrations or standard was mixed with 50 μL of 0.3 mM DPPH, and the reaction mixture was incubated at room temperature for 30 min in the dark. Absorbance was read at 517 nm. 

The scavenging activity was calculated using the following formula:

$$Free\;radical\;scavenging\;activity\;\left(\%\right)=\frac{\left(Absorbance\;of\;control-Absorbance\;of\;sample\right)}{Absorbance\;of\;control\;}\;\;\;\times 100\;$$

For FRAP activity, 50 μL of the sample’s concentrations or 240 μg/mL of gallic acid was mixed with 50 μL of 1% potassium ferricyanide (in 0.2 M sodium phosphate buffer, pH 6.6) at 50 °C for 30 min. Fifty μL of 10% TCA, and distilled water, and 10 μL of 0.1% ferric chloride were added to the reaction mixture. Absorbance was read at 700 nm. FRAP was expressed as percentage of 240 μg/mL gallic acid.

### 4.3. Inhibition of Carbohydrate Digestive Enzymes

The in vitro antidiabetic properties of the sample were determined by investigating its inhibitory effect on intestinal α-glucosidase and pancreatic α-amylase activities [50].

i. α-glucosidase activity

Briefly, 50 µL of the sample was mixed with 100 µL of 1.0 U m/L α-glucosidase enzyme dissolved in phosphate buffer (100 mmol/L, pH 6.8) and incubated for 20 min at 37 °C. *p*-nitrophenyl-α-*D*-glucopyranoside (pNPG) solution (of 5 mmol/L, 50 µL) was added to the reaction mixture and incubated for another 30 min at 37 °C. Absorbance was subsequently read at 405 nm. The inhibitory activity was recorded as a percentage of the control lacking inhibitor.

ii. α-amylase activity

Briefly, 50 µL of the sample concentration or acarbose was mixed with 100 µL of porcine pancreatic α-amylase (2 U/mL) in phosphate buffer (100 mmol/L, pH 6.8) and incubated for 20 min at 37 °C. A quantity of 50 µL of 1% starch was added to the reaction mixture and further incubated for 1 h at 37 °C. A quantity of 200 µL of the color reagent, dinitrosalicylate (DNS), was subsequently added to the reaction mixture and boiled for 10 min. Absorbance was read at 540 nm. The inhibitory activity was calculated as the proportion of the control lacking inhibitor. 

### 4.4. Animals for Ex Vivo Studies

Five male Sprague Dawley albino rats (200–250 g) were collected and housed at the Animal House facility of the Department of Biochemistry, College of Medicine, University of Lagos, Nigeria under the approved protocol, CMUL/REC/00314. The animals were euthanized with halothane after overnight fasting. Their psoas muscles were harvested for ex vivo studies. 

### 4.5. Muscle Glucose Uptake

Glucose uptake was carried out using a previously published method with slight modifications [51]. Briefly, 0.5 g of freshly harvested psoas muscles was incubated in 8 mL of Krebs buffer containing 11.1 mM glucose and different concentrations of coconut water for 2 h at 37 °C under a condition of 5% CO_2_ and 95% oxygen. Control consisted of incubation without coconut water, while the standard antidiabetic drug was metformin. Aliquots were collected before and after incubation. Glucose concentrations of aliquots were measured with a Glucose (GO) Assay Kit according to the manufacturer’s manual. 

Glucose uptake was calculated using the formula:

$$Glucose\;uptake\;per\;gm\;of\;rat\;psoas\;muscle=\frac{GC1-GC2\;}{Weight\;of\;muscle\;tissue\;(g)}$$

where *GC*1 and *GC*2 are glucose concentrations (mg/dL) before and after incubation, respectively.

After incubation and collection of buffer aliquots, the muscle tissues were collected and rinsed in normal saline. They were homogenized in cold phosphate buffer solution containing 1% triton X-100 (50 mM; pH 7.5). The homogenized tissues were centrifuged for 10 min at 15,000 rpm and 4 °C. The supernatants were collected and stored in 2 mL Eppendorf tubes at −20 °C until subsequent biochemical analyses.

### 4.6. Determination of Oxidative Stress Markers

Oxidative stress parameters were determined in the tissue supernatants by analyzing the reduced glutathione (GSH) [52] level, superoxide dismutase (SOD) [53] and catalase [54] activities, and malondialdehyde (MDA) [55] level.

i. Reduced glutathione (GSH) level

Briefly, 300 μL of the tissue supernatant was mixed with 10% TCA and centrifuged at 3500 rpm for 5 min (25 °C). Subsequently, 200 μL of the resulting supernatant was mixed with 50 μL of Ellman’s reagent in a 96-well plate, and allowed to stand on ice for 5 min. Absorbance was read at 415 nm. A GSH standard curve was used to extrapolate GSH level.

ii. Superoxide dismutase (SOD) enzyme activity

Briefly, 15 μL of the supernatant was mixed with 170 μL of 0.1 mM diethylenetriaminepentaacetic acid (DETAPAC) in a 96-well plate. Subsequently, 15 μL of 1.6 mM 6-hydroxydopamine (6-HD) was added to the reaction mixture. Absorbance was read at 492 nm wavelength for 3 min at 1 min intervals.

iii. Catalase enzyme activity

Briefly, 100 μL of the supernatant was mixed with 1000 μL H_2_O_2_ (65 μM) in 6.0 mM sodium phosphate buffer (pH 7.4). The reaction mixture was subsequently incubated at 37 °C for 2 min. The reaction was stopped with 4 mL of 32.4 mM ammonium molybdate, and absorbance was read at 347 nm against a blank.

iv. Malondialdehyde (MDA) level

Briefly, 200 μL of the supernatant was mixed with 200 μL of 8.1% SDS solution, 750 μL of 20% acetic acid, and 2 mL of 0.25% thiobarbituric acid (TBA). The reaction mixture was boiled for 1 h. After cooling, 200 μL of the reaction mixture was pipetted into a 96-well plate and the absorbance was measured at 532 nm. TBARS extrapolated from the MDA standard curve were used to estimate lipid peroxidation.

### 4.7. Determination of Purinergic Enzymes Activities

Purinergic activities of the muscle tissues were determined by assaying for ATPase [56,57] and ectonucleotidase (E-NTPDase) [58,59] activities.

v. ATPase activity

Briefly, 200 μL of the supernatants was incubated with 200 μL of 5 mM KCl, 1300 μL of 0.1 M Tris-HCl buffer, and 40 μL of 50 mM ATP in a shaker for 30 min at 37 °C. The reaction was stopped with 1 mL of distilled water and ammonium molybdate. Freshly prepared 9% ascorbic acid was subsequently added to the reaction mixture and allowed to stand on ice for 10 min. Absorbance was measured at 660 nm.

vi. ENTPDase activity

Briefly, 20 μL of the supernatants was mixed with 200 μL of the reaction buffer (1.5 mM CaCl_2_, 5 mM KCl, 0.1 mM EDTA, 10 mM glucose, 225 mM sucrose, and 45 mM Tris-HCl). The reaction mixture was incubated at 37 °C for 10 min. A quantity of 20 μL of 50 mM ATP was added to the reaction mixture and further incubated for 20 min at 37 °C. The reaction was stopped by adding 200 μL of 10% TCA and 200 μL of 1.25% ammonium molybdate. Freshly prepared 9% ascorbic acid was added to the reaction mixture, and allowed to stand on ice for 10 min. Absorbance was read at 600 nm.

### 4.8. LC-MS Analysis of Coconut Water

In order to determine its phytochemical constituents, the coconut water was subjected to LC-MS (Shimadzu LCMS-2020 Single Quadrupole) analysis by injecting directly into the machine via a loop as previously described [60]. The operating parameters were: **Stop time:** 60 min; **Photodiode Array (PDA) sampling frequency**: 1.5625 Hz; **Operating mode**: low pressure gradient; **Pump A**: LC-2030 Pump; **Mobile Phase A, B, C, and D**: 0.1% formic acid, methanol, acetonitrile, and water respectively; **Flow rate:** 0.3000 mL/min; **Start and End wavelengths**: 220 and 400 nm respectively; **Oven and Maximum Temperatures**: 40 and 50 °C respectively; **Start and End time**: 0.00 and 60.00 min respectively; **Acquisition mode**: Scan; **Scan Speed**: 5000 u/s; **Polarity**: Positive; **Event Time**: 0.25 s; **Detector Voltage**: +0.00 kV; **Threshold**: 0; **Start and End *m/z***: 100.00 and 1000.00 respectively; **Interface**: ESI; **Drying Gas**: 15.00 L/min. 

The phytochemicals were identified by direct search and comparison of mass spectral (MS) data with those of the Food Metabolome Database (www.foodb.ca, accessed on 5 September 2023).

### 4.9. Molecular Docking Analysis

i. Protein Target Selection and Preparation

The three-dimensional structures of the two protein receptors, AKT-2 serine and PI-3 kinase, were retrieved from the Protein Data Bank (PDB) (www.pdb.org/pdb, accessed on 5 September 2023) using the PDB IDs 3DOE and 7JWZ, respectively. The PDB database contains the experimental protein and nucleic acid structures. Discovery Studio 2021 was then used to improve and prepare the protein for docking. The protein was converted into a developing receptor by removing the co-crystallized ligand and additional water molecules, followed by the addition of hydrogen and charges.

ii. Ligand Selections and Preparations

Using Chem 3D 20.0, six compounds were simulated and energy optimized (MM44D). These optimized structures were used in the molecular docking tests.

iii. Molecular Docking

The molecular docking study was completed to carry out computations of six molecules with four protein receptors using the Lamarckian Genetic Algorithm in Auto Dock 4.2. In brief, the selected ligands were docked using Python Prescription 0.8 and a collection of automated molecular docking tools named Auto Dock Vina 4.2 with the active sites AKT-2 serine and PI-3 kinase. The protein data bank, partial charge, and atom type a file (PDBQT) was generated from PDB files that had already been prepared as input. The specific target location of the enzyme for the receptor’s active site was determined using a grid box. The AKT-2 serine grid point’s X, Y, and Z dimensions were 40, 50, and 48, respectively, while the grid center’s corresponding numbers were 27.507, −22.526, and 31.423. The PI-3 kinase grid point’s X, Y, and Z dimensions were 40, 54, and 63, respectively, whereas the grid center’s corresponding numbers were 48.619, 15.525, and 34.405. The docking studies for six compounds yielded 10 configurations for each protein–ligand combination for manual comparison examination. Additionally, text files containing the dock scoring values were created. The conformation with the lowest binding energy (kcal/mol) was shown to be the best docking position. Discovery Studio 2021 was also utilized to look at the interactions between ligands and proteins.

### 4.10. Statistical Analysis

Data were subjected to one-way analysis of variance (ANOVA) and presented as mean ± SD. A statistically significant difference between means were obtained at *p* < 0.05 using Tukey’s HSD multiple range post hoc test. IBM Statistical Package for the Social Sciences (SPSS) for Windows, version 23.0 (IBM Corp., Armonk, NY, USA) was used to carryout statistical analyses.

## 5. Conclusions

Taken together, these results indicate the ability of coconut water to enhance glucose uptake, with concomitant improved antioxidative and purinergic activities. It also indicates the potential of coconut water to suppress postprandial hyperglycemia, thereby giving more credence to the antidiabetic properties of coconut water. These biological activities may be attributed to the synergistic effects of the LC-MS-identified compounds. However, in vivo and molecular studies are required to further decipher these mechanisms in diabetic models.

## Figures and Tables

**Table 1:** IC_50_ values of biological activities *.

Biological Activities	Coconut Water	Ascorbic Water
	(μg/mL)	
DPPH	34.09	0.09
FRAP	>1000	0.23
α-glucosidase	338.27	70.58
α-amylase	219.73	0.02

* IC_50_ (half-maximal inhibitory concentration) indicates the concentration of the extract needed to inhibit a biological process by half.

**Table 2:** Binding energy and bonds.

Protein	Compounds	Estimated Free Binding Energy (kcal/mol)	Estimated Inhibition Constant, Ki in nM	Hydrogen Bonds (Distance Å)	Electrostatic Interaction	Electrostatic Interaction
**3DOE**	Ellagic acid	−8.99	257.83	LYS29 (2.92223) THR30 (1.83044) THR30 (2.10013) THR31 (2.21939) THR31 (1.77066) ASP66 (1.74398) ASP66 (2.20146) ASN26 (2.19584) ASN26 (2.12805) ASN26 (2.24013) THR47 (3.12677)	LYS29	LYS29 PRO46 (x2)
	Butin	−8.35	760.49	ALA27 (2.46832) GLY28 (2.19884) LYS29 (2.25839) LYS29 (1.96312) THR30 (2.9807) GLN70 (1.91152) GLN70 (2.55934) THR47 (1.75311)		PRO46
	Quercetin	−7.5	3.16	GLY69 (2.12109) GLN70 (2.4799) LYS126 (2.15314) THR31 (1.97452) THR47 (2.13694) ASP25 (2.51937) GLY68 (3.52814) ASN26 (2.54491) THR47 (3.0409)	LYS29	PRO46 (x2)
	Protocatechuic acid	−6.13	32.35	GLY28 (2.08006) LYS29 (2.21339) LYS29 (1.89555) THR31 (1.93536) LEU24 (2.32277) ASP25 (3.15522)		
	Baicalin	−8.45	635.2	ALA27 (2.22248) GLY28 (2.09824) LYS29 (2.244) LYS29 (1.75644) LYS29 (1.7697) THR30 (2.91587) THR31 (2.25074) THR30 (3.00405) VAL160 (3.07995)		LYS126 VAL160 ALA159
	Silibinin	−8.48	605.62	THR43 (2.6353) ILE44 (2.08981) GLN70 (2.45455) ILE44 (1.98277) ILE41 (2.11285) LEU24 (2.59707) ASP25 (3.12523)	LYS126	ASN26 ALA27 PRO46 (x2) ILE44
**7JWZ**	Ellagic acid	−8.55	543.33	VAL882 (2.04853) ALA885 (2.11578) ASP964 (1.81248) ASP964 (2.2236)		MET953 (x2) ILE963 (x2) TRP812 TYR867 VAL882 ALA885 ILE831
	Butin	−7.07	6.55	VAL882 (1.79667) GLU880 (1.98775) GLU880 (2.09533)		ILE831 MET804 MET953 PRO810 ILE831 ILE963
	Quercetin	−7.25	4.88	VAL882 (1.74091) ASP964 (2.09705) GLU880 (2.10031) ASP964 (2.12019)		ILE879 ILE963 (x2) MET953 (x2) TRP812 TYR867 ILE831
	Protocatechuic acid	−5.17	162.78	ASP964 (2.07765) GLU880 (2.25826) ASP964 (2.00965) ASP964 (1.87388)		ALE879 TYR867 ILE831 ILE963
	Baicalin	−8.46	630.16	ALA885 (2.80904) ALA885 (1.89965)		MET953 (x3) TRP812 (x2) TYR867 ALA885 VAL882 ILE963
	Silibinin	−9.03	240.83	LYS802 (1.80046) TRP812 (2.2991) ASP964 (1.7375) ASP964 (1.85756) GLU880 (2.2275) PRO810 (2.2449)		LYS802 ILE963 TRP812 MET804 PRO810 ILE831(x2) ILE879

## Data Availability

All data presented in the article.
